# Integrated multi-omics analysis revealed the response mechanism of *Osmanthus fragrans* leaves against the infection by *Botryosphaeria dothidea*

**DOI:** 10.3389/fpls.2026.1830904

**Published:** 2026-05-15

**Authors:** Shizhen Wang, Mingjiang Li, Jiaoyu Wang, Na Tian, Jun Huang, Juan Zhang

**Affiliations:** 1 Institute of Garden Plants & Flowers, Zhejiang Academy of Agricultural Sciences, Hangzhou, China; 2 State Key Laboratory for Quality and safey of Agro-Products, Zhejiang Provincial Key Laboratory of Agricultural Microbiomics, Key Laboratory of Agricultural Microbiome (MARA), Institute of Plant Protection and Microbiology, Zhejiang Academy of Agricultural sciences, Hangzhou, China; 3 General Office, Agricultural Technology Extension Service Center of Yinchuan, Yinchuan, Ningxia, China; 4 State Key Laboratory for Quality and Safety of Agro-products, Institute of Plant Protection and Microbiology, Zhejiang Academy of Agricultural Sciences, Hangzhou, China

**Keywords:** *Botryosphaeria dothidea*, metabonomics, *Osmanthus fragrans*, response mechanism, transcriptome metabolism

## Abstract

*Osmanthus fragrans Lour. (Oleaceae)*, a perennial woody species, faces severe threats from *Botryosphaeria dothidea*, which causes severe diseases in economically important woody plants, leading to substantial agricultural losses. However, the stage-specific molecular and metabolic mechanisms underlying osmanthus resistance to *B. dothidea* remain unclear. Here, we performed integrated transcriptomic and metabolomic analyses combined with Weighted Gene Co-expression Network Analysis (WGCNA) to systematically clarify the dynamic defense responses of the previously screened resistant genotype 'No. 35' and susceptible genotype 'No. 37' at early (0 days post inoculation, dpi), middle (3 dpi), and late (7 dpi) stages of *B. dothidea* infection. A total of 30,125 DEGs and 356 DAMs were identified, respectively. WGCNA revealed that stage-specific defense modules with hub genes from ERF5, RVE and WRKY transcription factor families were significantly correlated with disease resistance at early, middle and late infection stages respectively, forming a sequential multi-layered defense regulatory network. Metabolome profiling revealed the DAMs enriched in flavonoid biosynthesis with significantly upregulated *4CL* and *F3H* genes and enriched luteolin/apigenin and phenylpropanoid synthesis metabolism at 7dpi in enhancing the tolerance of *B. dothidea* infection. Our research spotlights the genes *4CL, F3H*, and accumulation of luteolin/apigenin, which showed a strong positive correlation. Its defense mechanism primarily functions by upregulating key genes in the flavonoid biosynthesis, which drives the accumulation of defensive metabolites, and ultimately leading to the enrichment of flavonoid precursors in leaves. These findings provide a theoretical basis for understanding the multi-omics synergistic mechanism of biotic stress resistance, molecular breeding, and biological control, offering theoretical support for sustainable disease management in Osmanthus cultivation.

## Introduction

*Osmanthus fragrans* Lour. (Oleaceae), a perennial woody species native to Asia, with subsequent introduction to Europe (Wu et al., 2022), possesses significant cultural and economic importance in East Asia. Commonly termed sweet osmanthus, this species is extensively cultivated in China for its aromatic flowers. These flowers contain essential oils (e.g., linalool and benzyl acetate) and bioactive compounds (e.g., flavonoids and triterpenoids), which are utilized in traditional medicine, cosmetics, and food industries (Zheng et al., 2009). Beyond ornamental value, *O. fragrans* provides critical ecosystem services, including carbon sequestration and air pollutant mitigation, with global commercial value.

However, *O. fragrans* faces severe threats from *Botryosphaeria dothidea*, a hemibiotrophic ascomycete pathogen with over 200 host species, including economically important crops like apples (*Malus pumila*), pears (*Pyrus*), and kiwifruit (*Actinidia deliciosa*), causing devastating disease (Marsberg et al., 2017). Severe outbreaks in endemic regions may incur 80% production loss, threatening the development of the fruit industry and forestry ecosystem (Zhang et al., 2021). In *O. fragrans*, leaf dieback caused by *B. dothidea* was first reported in 2010 (Xie et al., 2010), characterized by necrotic lesions expanding from leaf margins to the entire blade, leading to reduced photosynthetic efficiency, flower yield decline, and even plant mortality (Hao et al., 2022; Fan et al., 2021). The sweet osmanthus is widely cultivated in Southern China and has been selected as the city flower in more than 20 cities (Xie et al., 2010). However, *B. dothidea* has become a devastating pathogen of *O. fragrans* in recent years, breaking out not only in Nanning City but also in Zhengzhou, Henan Province. Xie et al. (2010) reported that 97.5% osmanthus leaves were infected by *B. dothidea* with a disease index of 59. Currently, the control of *B. dothidea* on *O. fragrans* mainly relies on pruning of infected tissues and benzimidazole fungicides, but pruning is labor-intensive and only effective for mild infections, failing to prevent the spread of the pathogen in large-scale plantations, and prolonged application has induced fungicide resistance development (Zhang et al., 2021) and environmental contamination, thereby posing substantial risks to non-target organisms, food safety, and human health (Fan et al., 2021). Additionally, there are no commercially resistant cultivars of *O. fragrans* available, as the molecular mechanisms underlying *O. fragrans* resistance to *B. dothidea* remain poorly understood. Thus, understanding the molecular basis of *O. fragrans* resistance to *B. dothidea* is critical for implementing sustainable disease management. Consequently, screening resistant germplasms from existing *O. fragrans* collections for breeding and mechanistic studies is imperative.

Plant defense mechanisms involve complex transcriptional and metabolic reprogramming with multi-tiered regulation, which cannot be fully resolved by single-omics alone. Integrated multi-omics analysis overcomes the limitations of single-omics approaches, enabling systematic dissection of the regulatory networks underlying plant defense responses (Mengiste and Liao, 2025). Numerous studies have applied integrated multi-omics analysis to identify crop disease resistance mechanisms (He et al., 2018; Zhang et al., 2019; Liu et al., 2023).

Previous studies on *B. dothidea* pathogenesis have primarily focused on its genome architecture, mating strategies, and host penetration mechanisms (Nagel et al., 2018; Yu et al., 2022; Yang et al., 2025). However, the molecular mechanisms underlying *O. fragrans* resistance to *B. dothidea* remain poorly characterized. This knowledge gap significantly limited the development of targeted genetic breeding strategies and sustainable disease management protocols. Therefore, we aimed to clarify the defensive mechanisms of *O. fragrans* against *B. dothidea* infection using integrated transcriptomic and metabolomic approaches.

In this study, we phenotypically characterized a total of 50 *O. fragrans* accessions based on disease severity indices to identify resistant and susceptible materials. By integrating transcriptomic and metabolomic analyses between resistant and susceptible germplasms, we aimed to decipher the regulatory dynamics of differentially expressed genes (DEGs) and differentially accumulated metabolites (DAMs), as well as the underlying molecular regulatory networks, during *B. dothidea* infection. Furthermore, through DEG and co-expression network analyses, we sought to identify hub genes that exert pivotal functions at distinct stages of fungal infection. These results present insight into the molecular regulatory mechanisms of the defense response of *O. fragrans* to *B. dothidea* inoculation.

## Results

### Evaluation of the sensitivity of 50 *O. fragrans* germplasms against *B. dothidea*

Fifty *O. fragrans* germplasms were inoculated with *B. dothidea* mycelial plugs on leaves and incubated at 28 °C. Infection types of leaves from each accession were evaluated and recorded at 7 days post-inoculation (dpi).

**Figure 1:**
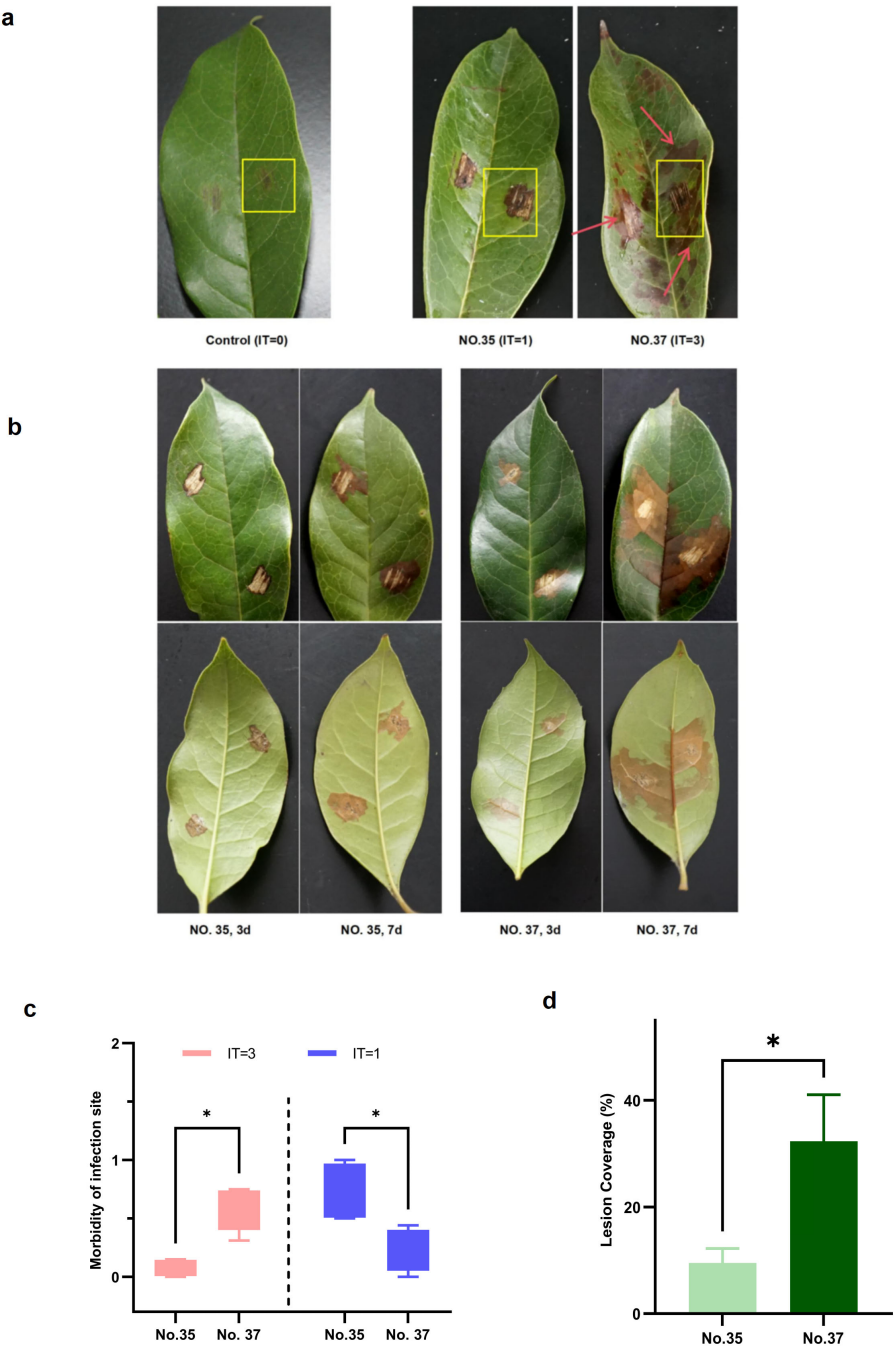
Comparative analysis of *Osmanthus fragrans* accessions response to *Botryosphaeria dothidea* infection at 7 days post-inoculation (dpi). **(a)** Phenotypes of resistant and susceptible *O. fragrans* accessions against *B. dothidea* screened from 50 *O. fragrans* material resources. Control, mock inoculation with sterile Potato Dextrose Agar (PDA). **(b)** Phenotypes of resistant accession No. 35 and susceptible accession No. 37 leaves at 3 and 7 dpi, respectively. **(c)** Morbidity of infection site of accession No. 35 and No. 37 with IT = 1 and IT = 3, respectively (*p < 0.05, two-way ANOVA; IT, infection type). **(d)** Leaf lesion coverage of accession No. 35 and No. 37 leaves. Asterisks indicate statistically significant differences (*p < 0.05, *t*-test).

To characterize the pathogenic sensitivity of 50 *O. fragrans* germplasms, morbidity at infection sites was quantified. Based on the infection grading system (detailed in Materials and Methods), accession No. 35 exhibited significant resistance with an infection type (IT) of 1, characterized by localized lesions restricted to the mycelial plug area (**Figure 1a**). In contrast, accession No. 37 displayed susceptibility with an IT of 3, featuring lesions rapidly expanding to leaf margins and higher lesion density. These accessions were selected as resistant and susceptible references (**Figure 1c**). Based on the screening criteria, IT = 1 (localized lesions) was defined as resistance, and IT = 3 (systemic expansion) as susceptibility, thereby designating No. 35 and No. 37 as reference accessions for resistant and susceptible phenotypes, respectively.

### Phenotype and RNA-seq of resistant/susceptible accessions at different incubation times

To elucidate the resistance divergence of *O. fragrans* accessions against *B. dothidea*, leaves of accessions No. 35 and No. 37 were wound-inoculated and incubated in darkness in humid trays at 28 °C, with sampling at 0, 3, and 7 dpi. The inoculation experiment was conducted three times. Results demonstrated that both accessions developed localized lesions with comparable areas at 3 dpi. However, No. 37 exhibited rapid lesion expansion occupying ~36% of total leaf area at 7 dpi (**Figures 1b, d**) and aggressively colonized healthy tissues toward leaf margins at 7 dpi. In contrast, No. 35 showed constrained pathogenesis, with lesions extending only ~2 mm beyond inoculation sites and covering merely ~9.3% of leaf area (**Figures 1b–d**). These findings confirm that at 28 °C, No. 35 maintains stable resistance, which shows a low lesion expansion rate, while No. 37 displays high susceptibility and sensitivity against *B. dothidea*. Overall, in the condition of 28 °C, lesions developed slowly in the initial period in both accessions at 3 dpi, but lesions developed rapidly in the susceptible accession and were still slow in the resistant accession.

### Transcriptome profiling of resistant and susceptible accessions in response to *B. dothidea* infection

To characterize transcriptional dynamics underlying resistance/susceptibility in *O. fragrans* during *B. dothidea* infection, resistant (No. 35) and susceptible (No. 37) accessions were wound-inoculated and incubated at 28 °C with leaf sampling at 0, 3, and 7 dpi.

RNA sequencing generated a total of ~94.5 Gb of clean data. After quality filtering, the average Q20 and Q30 values were above 98.3% and 94.3%, respectively, with an average Guanine-Cytosine (GC) content of 43% (**Supplementary Table 1**). *De novo* assembly yielded a final set of 75, 851 unigenes, with an N50 length of 1, 818 bp, an average length of 1, 063 bp (**Supplementary Table 2**), and a Benchmarking Universal Single-Copy Orthologs (BUSCO) score of approximately 68%. Each sample produced 14.8–16.9 Gb of clean data, with 80.44%–86.75% of clean reads uniquely mapped to the assembled unigene set, confirming the high quality and reliability of the transcriptome data for downstream analysis.

**Figure 2:**
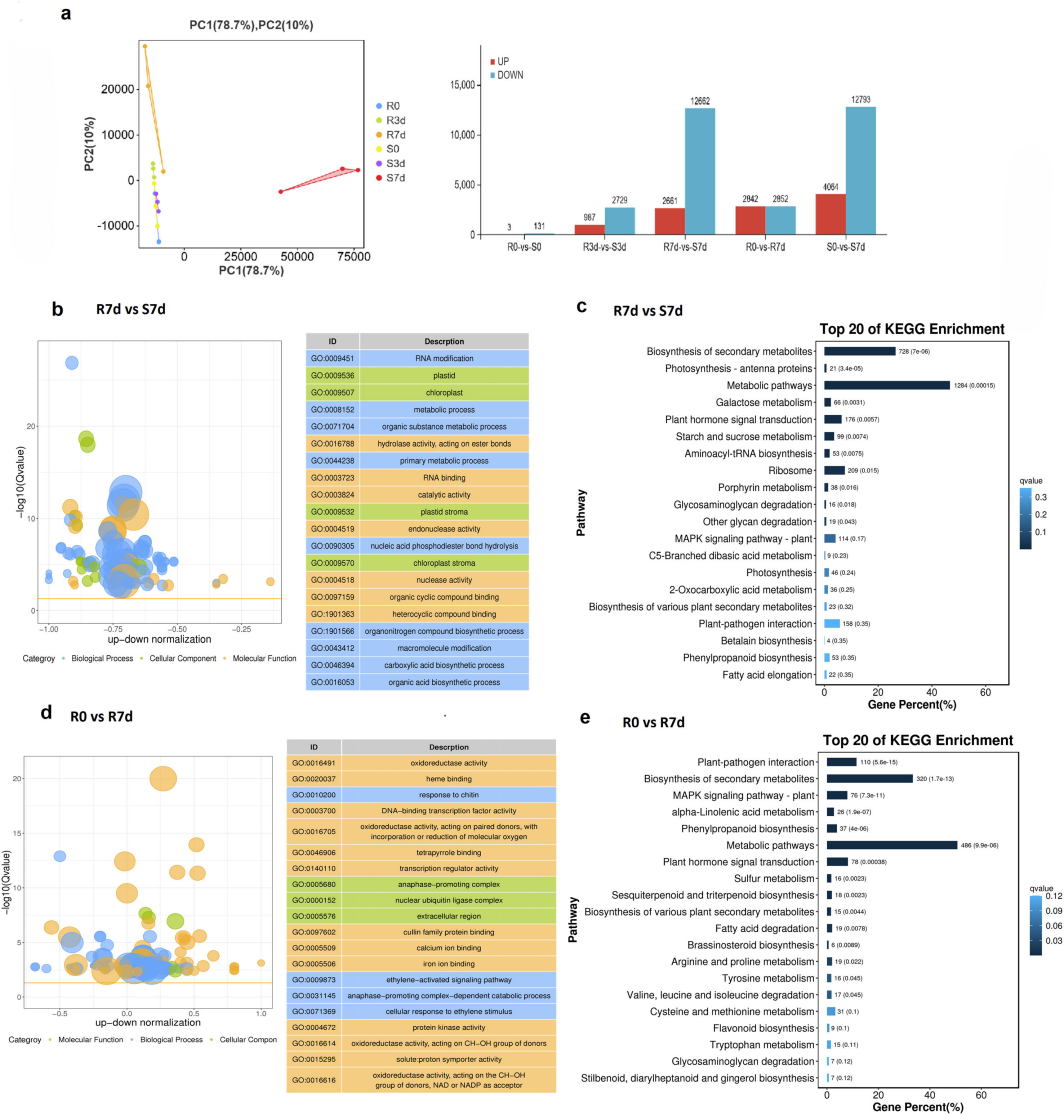
Transcriptional dynamics of differentially expressed genes (DEGs) in two *Osmanthus fragrans* accessions in response to *Botryosphaeria dothidea* infection. **(a)** Principal component analysis (PCA) showing the overall transcriptomic clustering and repeatability of all samples, along with the number statistics of DEGs identified from the five comparative groups (R0 vs S0, R3d vs S3d, R7d vs S7d, R0 vs R7d, and S0 vs S7d). **(b)** Bubble plot of the top 20 significantly enriched Gene Ontology (GO) terms for the R7d vs S7d comparison. **(c)** Bar plots of the top 20 significantly enriched Kyoto Encyclopedia of Genes and Genomes (KEGG) pathways for the late infection phase comparison between R7d vs S7d. **(d)** Bubble plot of the top 20 significantly enriched GO terms for the R0 vs R7d comparison. **(e)** Bar plots of the top 20 significantly enriched KEGG pathways for the time–course comparison within the resistant accession No. 35, R0d vs R7d. R represents the resistant *O. fragrans* accession No. 35, and S represents the susceptible *O. fragrans* accession No. 37. The number following R/S indicates the sampling time point (0, 3, and 7 days) post-inoculation. For all GO bubble plots, Y = −log_10_ (Q value), X = z-score; yellow line = Q = 0.05 threshold. Top 20 Q-ranked GO terms are on the right; colors mark GO ontologies: biological process (blue), cellular component (green), and molecular function (orange). For all KEGG bar plots, Y = top 20 KEGG pathways (Q value), X = percent of annotated DEGs/total DEGs per group. Bar labels: enriched DEG count and corresponding Q value.

Principal component analysis (PCA) was performed on the gene expression matrix normalized by Fragments Per Kilobase of transcript per Million mapped reads (FPKM) (Mortazavi et al., 2008). The results showed a clear separation between the resistant (No. 35; R) and susceptible (No. 37; S) accessions along principal component 1 (PC1) (**Figure 2a**), indicating fundamental divergence in the genome-wide transcriptional reprogramming between the two genotypes in response to pathogen stress.

To further clarify the transcriptomic variation between the two accessions, DEG analysis was performed across five pairwise comparative groups: R0 vs S0, R3d vs S3d, R7d vs S7d, R0 vs R7d, and S0 vs S7d. Comparative analysis revealed basal transcriptomic difference between the two accessions at 0 dpi; an increase in DEG number was detected at 3 dpi, followed by a dramatic amplification at 7 dpi (**Figure 2a**). Similarly, a large number of DEGs were identified in the time–course comparisons between 7 and 0 dpi, R0d vs R7d group and S0d vs S7d group (**Figure 2a**). These results demonstrated a time-dependent amplification of transcriptomic divergence between the resistant and susceptible accessions, which peaked at the late infection stage (7 dpi).

To elucidate the biological functions of the identified DEGs, we performed Gene Ontology (GO) enrichment and Kyoto Encyclopedia of Genes and Genomes (KEGG) pathway enrichment analysis for the five comparative groups. For the comparative groups (R0 vs S0, R3d vs S3d, and R7d vs S7d), the top 20 enriched GO terms in the group of R0d vs S0d were mainly associated with oxidoreductase activity, monooxygenase activity, cell recognition, and defense response, which may reflect the differential wound response of the two accessions immediately after inoculation (**Supplementary Figure 1a**). The R3d vs S3d group was significantly enriched in GO terms related to DNA integration, DNA-binding transcription factor activity, oxidoreductase activity, and transcription regulator activity (**Supplementary Figure 1b**), while the R7d vs S7d group was dominated by terms associated with RNA modification, metabolic process, hydrolase activity, and catalytic activity (**Figure 2b**). For the time–course groups, the susceptible accession (S0 vs S7d) showed significant enrichment of chloroplast-related terms (thylakoid and plastid) and carboxylic acid metabolism (**Supplementary Figure 1c**). In contrast, the resistant accession (R0d vs R7d) exhibited sustained enrichment of terms related to ethylene signaling (ethylene-activated signaling pathway and cellular response to ethylene) and response to chitin (**Figure 2d**), suggesting a potential role of ethylene in the defense response of the resistant genotype.

**Figure 3:**
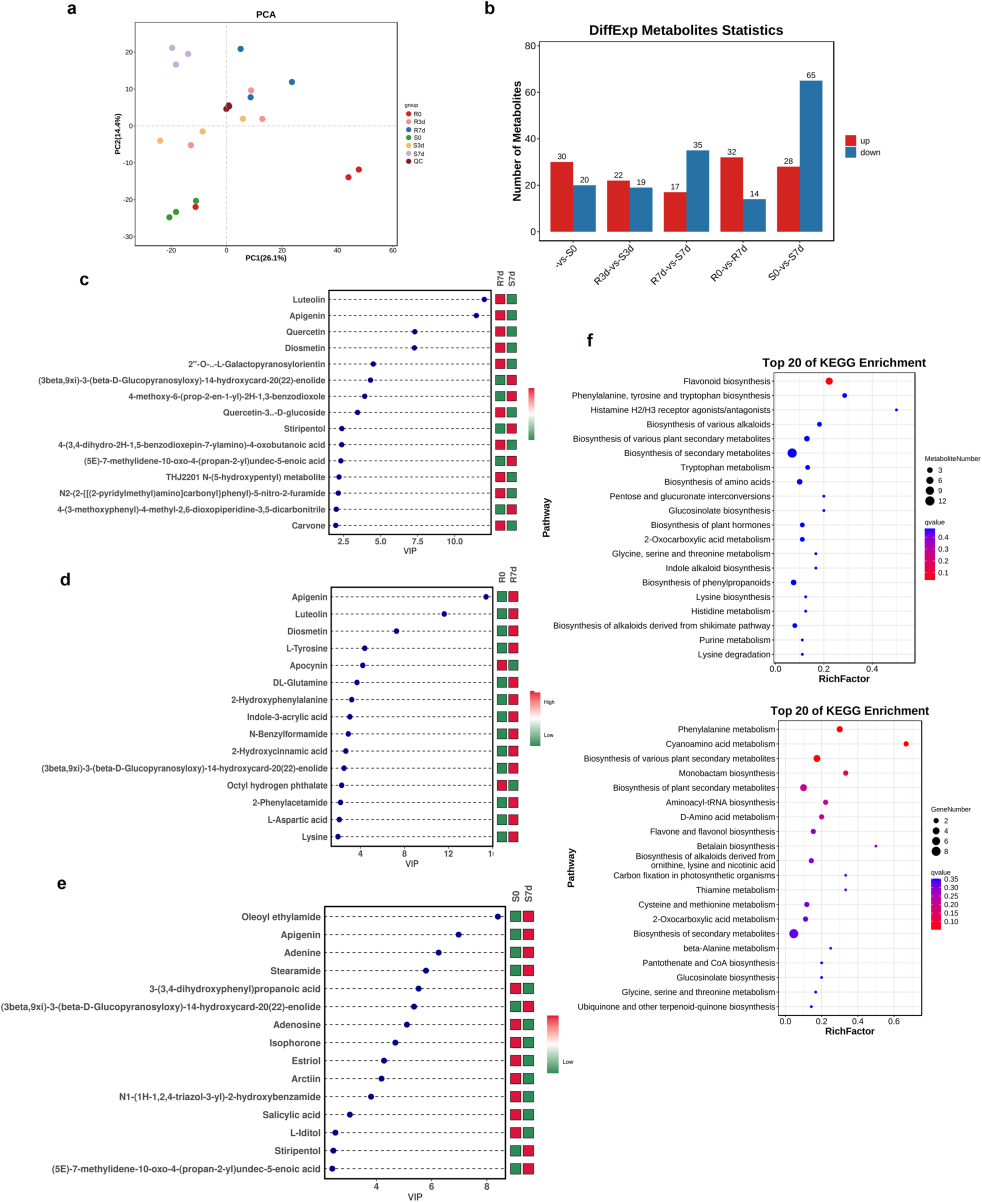
Metabolomic profiling and differentially accumulated metabolite (DAM) analysis of resistant and susceptible *Osmanthus fragrans* accessions in response to *Botryosphaeria dothidea* infection across pairwise comparative groups. **(a)** Principal component analysis (PCA) showing the overall metabolic profile clustering and biological repeatability of all samples. **(b)** Quantitative statistics of upregulated and downregulated DAMs identified from the five core pairwise comparative groups. **(c)** Variable Importance in Projection (VIP) plot derived from the orthogonal partial least squares discriminant analysis (OPLS-DA) model for the R7d vs S7d comparative group. **(d)** VIP plot derived from the OPLS-DA model for the R0d vs R7d comparative group. **(e)** VIP plot derived from the OPLS-DA model for the S0d vs S7d comparative group. **(f)** Bar plots of the top 20 significantly enriched Kyoto Encyclopedia of Genes and Genomes (KEGG) pathways for the R3d vs S3d and R0d vs R7d comparative groups. DAMs were defined as metabolites with p-value of *t*-test <0.05 and VIP ≥ 1. R represents the *B. dothidea*-resistant *O. fragrans* accession No. 35, and S represents the susceptible *O. fragrans* accession No. 37. The number following R/S indicates the sampling time point (0, 3, and 7 days) post-inoculation. OPLS-DA was performed to identify metabolites contributing significantly to intergroup differences. Red indicates abundance accumulation, and green indicates less accumulation.

Pathway enrichment profiling further revealed the distinct defense pathway activation patterns between the two accessions. Specifically, the biosynthesis of secondary metabolites and the MAPK signaling pathway were enriched in the R0d vs S0d group (**Supplementary Figure 1a**). The R3d vs S3d group was dominated by phenylpropanoid biosynthesis and plant hormone signal transduction (**Supplementary Figure 1b**), while the R7d vs S7d group showed coordinated activation of plant–pathogen interaction, MAPK signaling pathway, phenylpropanoid biosynthesis, and hormone signaling pathways (**Figure 2c**). For the time–course groups, the resistant accession (R0 vs R7d) was significantly enriched in plant–pathogen interaction, phenylpropanoid biosynthesis, and MAPK signaling pathway (**Figure 3e**), whereas the susceptible accession (S0d vs S7d) was mainly enriched in general metabolic pathways, the biosynthesis of secondary metabolites, and plant hormone signal transduction (**Supplementary Figure 1c**). Collectively, these findings indicated that the resistant accession mounts a targeted and effective defense response via the activation of specific immune signaling pathways and defensive secondary metabolite biosynthesis upon *B. dothidea* infection. In contrast, the susceptible accession triggers a broad but non-specific metabolic and hormonal response, which fails to establish effective resistance against pathogen invasion.

### Metabolome analysis of *O. fragrans* leaves responsive to *B. dothidea* infection

Non-targeted metabolomics analysis was performed in resistant (R) and susceptible (S) leaves at 0, 3, and 7 dpi and identified 1, 510 metabolites with 282 significantly regulated DAMs across five comparisons, designated as R0 vs S0, R3d vs S3d, R7d vs S7d, R0 vs R7d, and S0 vs S7d (PCA; **Figures 3a, b**).

Overall, metabolomics identified 50 significantly accumulated DAMs in R0 vs S0 (30 up/20 down) (**Supplementary Table 3**), 41 DAMs in R3d vs S3d (22 up/19 down) (**Supplementary Table 4**), and 52 DAMs in R7d vs S7d (17 up/35 down) (**Supplementary Table 5**); resistant time-series (R0 vs R7d) contained 46 DAMs (32 up/14 down) (**Supplementary Table 6**), while susceptible progression (S0 vs S7d) showed 93 DAMs (28 up/65 down) (**Supplementary Table 7**, **Figure 3b**). These demonstrate metabolic dysregulation in the susceptible accession, whereas the resistant genotype maintains homeostasis through the precision regulation of core defense metabolites (e.g., flavonoids), with significantly lower DAMs increment (R0 vs R7d) than the susceptible group.

Metabolomics integrated with KEGG enrichment characterized key metabolite classes (benzenoids, phenylpropanoids, organic acids, etc.). Variable Importance in Projection (VIP) value indicates the importance of variables and their contribution to sample discrimination, and the top 15 DAMs with VIP value are shown in **Figures 3c–e**. Flavonoid DAMs, such as luteolin, apigenin, and quercetin, were massively accumulated in R7d compared with S7d (**Figure 3c**). Similarly, when compared with that in R0, the accumulation of luteolin and apigenin was enhanced in R7d; moreover, amino acids (e.g., tyrosine, glutamine, and lysine) were significantly enriched in R7d (**Figure 3d**). Compared with those in the group of R0 vs S0, the contents of luteolin and apigenin were significantly elevated, and their accumulation was specifically induced by *B. dothidea* infection (**Supplementary Figure 2**). In the S0 vs S7d comparison, salicylic acid (SA) content showed a marked decrease in S7d relative to S0 (**Figure 3e**), weakening Pathogenesis-Related (PR) gene induction (Han et al., 2022; Hu et al., 2024). KEGG enrichment showed R0-R7d DAMs enriched in phenylalanine metabolism, R3d–S3d in tryptophan metabolism and flavonoid biosynthesis (“tryptophan metabolism”, “glucosinolate biosynthesis”, “flavonoid biosynthesis”, “glycine, serine and threonine metabolism”, and “indole alkaloid biosynthesis” pathways), and R7d–S7d in flavone/flavonol biosynthesis (“flavonoid biosynthesis” and “flavone and flavonol biosynthesis) (**Figure 3f**).

**Figure 4:**
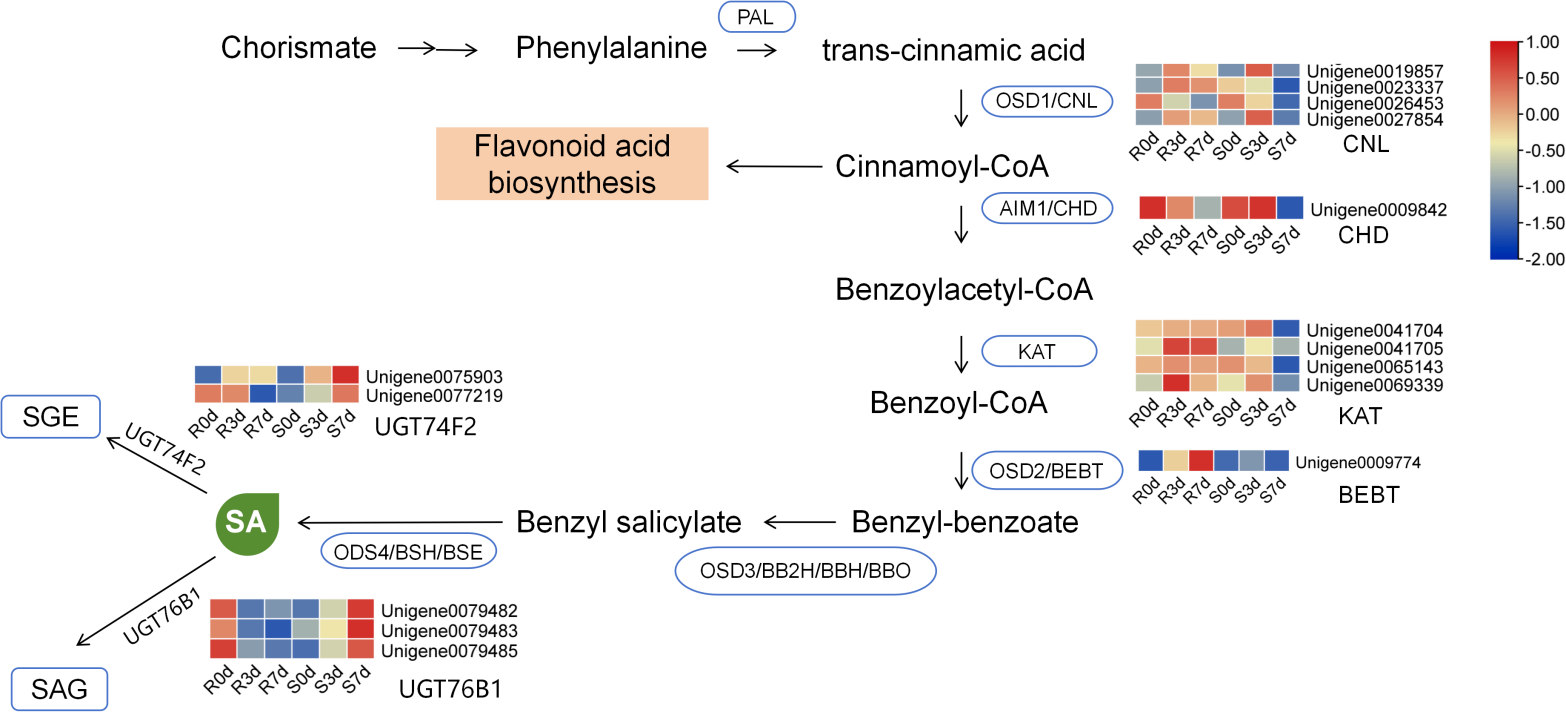
Regulatory network of the salicylic acid (SA) biosynthesis pathway in resistance and susceptible *Osmanthus fragrans* response to *Botryosphaeria dothidea* infection at 0, 3, and 7 dpi.

Numerous studies have demonstrated that changes in endogenous SA content directly reflect the activity of the SA-mediated defense pathway and that reduced SA accumulation is consistently and significantly associated with increased plant susceptibility to pathogen infection. In this study, we first confirmed that the endogenous SA content of the susceptible material No. 37 showed a significant decrease at 7 dpi. To further elucidate the molecular mechanism underlying this phenotype, we systematically analyzed transcriptomic expression profiles of key genes involved in SA biosynthesis and metabolic pathways and generated a heatmap of their expression levels (**Figure 4**). The results revealed that the core genes in the SA biosynthesis pathway, including CNL, CHD, KAT, and BEBT, were all significantly downregulated in the susceptible group (No. 37) at 7 dpi (S7d). This downregulation directly resulted in insufficient capacity of endogenous SA biosynthesis in the susceptible material. Meanwhile, the UGT genes responsible for catalyzing the glycosylation modification of SA and its conversion into inactive SA glucoside (SAG) and SA glucose ester (SGE), namely, UGT74F2 and UGT76B1, were significantly upregulated in the S7d group. This expression pattern led to rapid and continuous glycosylation and inactivation of the small amount of free SA synthesized in the plants, resulting in insufficient supply and excessive consumption of biologically active free SA in No. 37.

The integrated transcriptomic and metabolomic results presented here delineate the contrasting defense strategies of resistant and susceptible *O. fragrans* accessions against *B. dothidea* infection. Specifically, the resistant genotype mounts an effective two-stage defense response, characterized by the rapid biosynthesis of defense-related precursors at the early infection stage (3 dpi), and the subsequent establishment of a robust chemical barrier at the late infection stage (7 dpi) through the synergistic regulation of flavonoid and amino acid metabolism. By contrast, the functional disruption of the SA biosynthesis and metabolic cascade, which leads to the complete breakdown of SA-mediated defense signaling, is identified as the core molecular mechanism responsible for the severely impaired disease resistance in the susceptible accession.

### RT-PCR validation of DEGs

**Figure 5:**
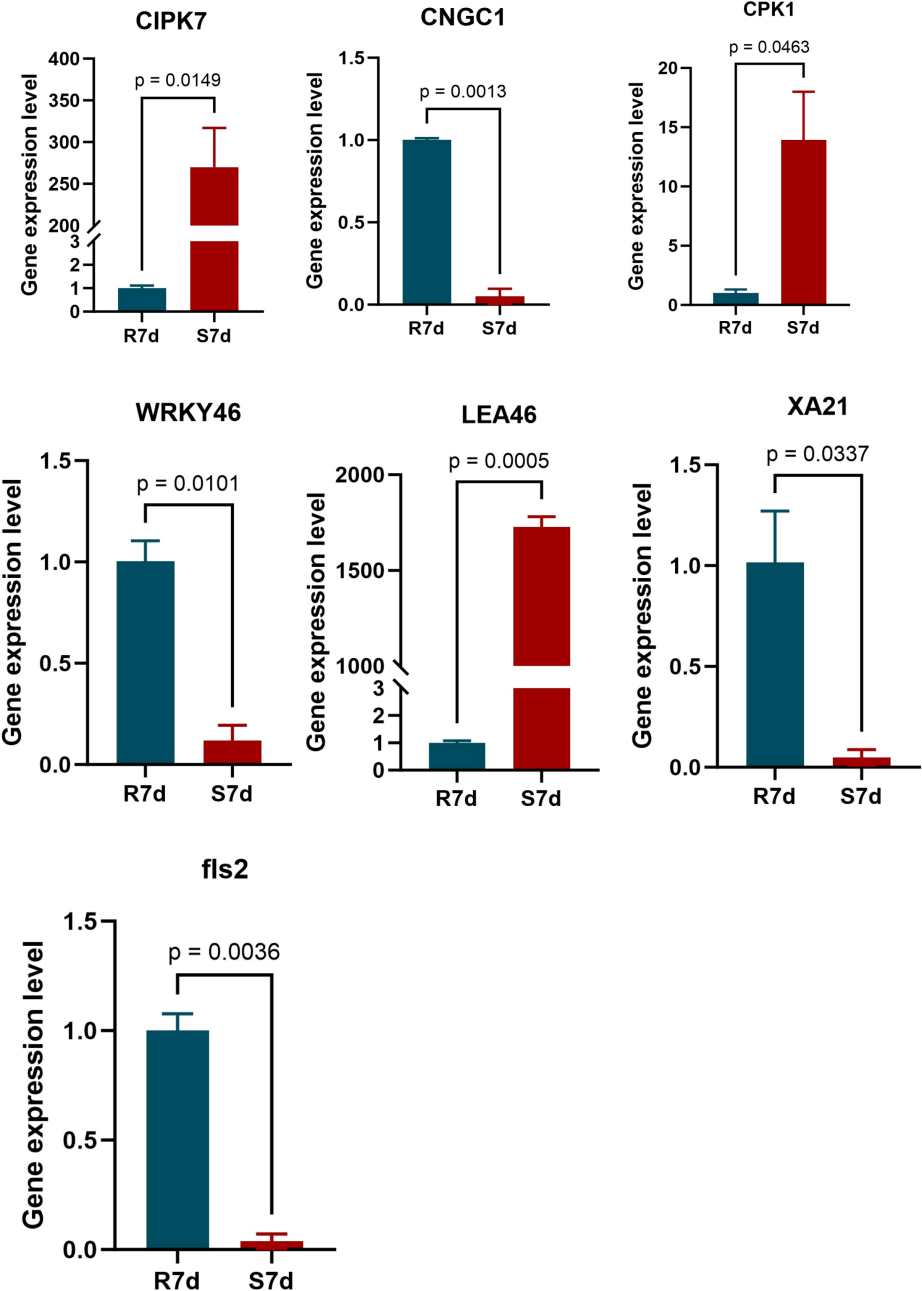
Quantitative real-time PCR (qRT-PCR) validation of differentially expressed genes (DEGs) identified via RNA-seq. The relative transcript levels of representative defense-related DEGs were determined via qRT-PCR in leaf samples of *Botryosphaeria dothidea*-resistant *Osmanthus fragrans* accessions No. 35 and susceptible *O. fragrans* accessions No. 37 collected at 7 dpi. The *ACT* gene was used as the stable internal reference for transcript level normalization. Error bars represent the standard deviation (SD) of three independent biological replicates. Statistical significance was assessed using *t*-test, and p-values are shown for each gene.

To confirm transcriptome reliability, RT-PCR was conducted on six significantly differentially expressed genes (*CIPK7*, *LEA46*, *WRKY46*, *FLS2*, *XA21*, and *CPK1*) using *ACT* as a reference gene. The expression levels of the selected DEGs at 7 dpi were consistent with RNA-seq results. The relative expression patterns of the selected DEGs at 7 dpi with *B. dothidea* were highly consistent with the RNA-seq results, confirming the reliability of our transcriptome profiling. Among these genes, *CIPK7*, *LEA46*, and *CPK1* were significantly upregulated in the leaves of the susceptible *O. fragrans* accession. In contrast, the transcription factor gene *WRKY46* and the immune receptor gene XA21 were highly expressed in the resistant accession, highlighting the functional conservation of core plant disease resistance pathways in woody plant species (**Figure 5**).

### Correlation analysis of DEGs and DAMs

To further understand the network of transcripts and metabolic processes, we integrated the DEGs and DAMs data. Pathway, bidirectional orthogonal projections to latent structures (O2PLS) modeling, and Pearson’s model were constructed for the two data matrices and used to illuminate the internal relationship and correlation degree between the two omics caused by the main genes and metabolites.

**Figure 6:**
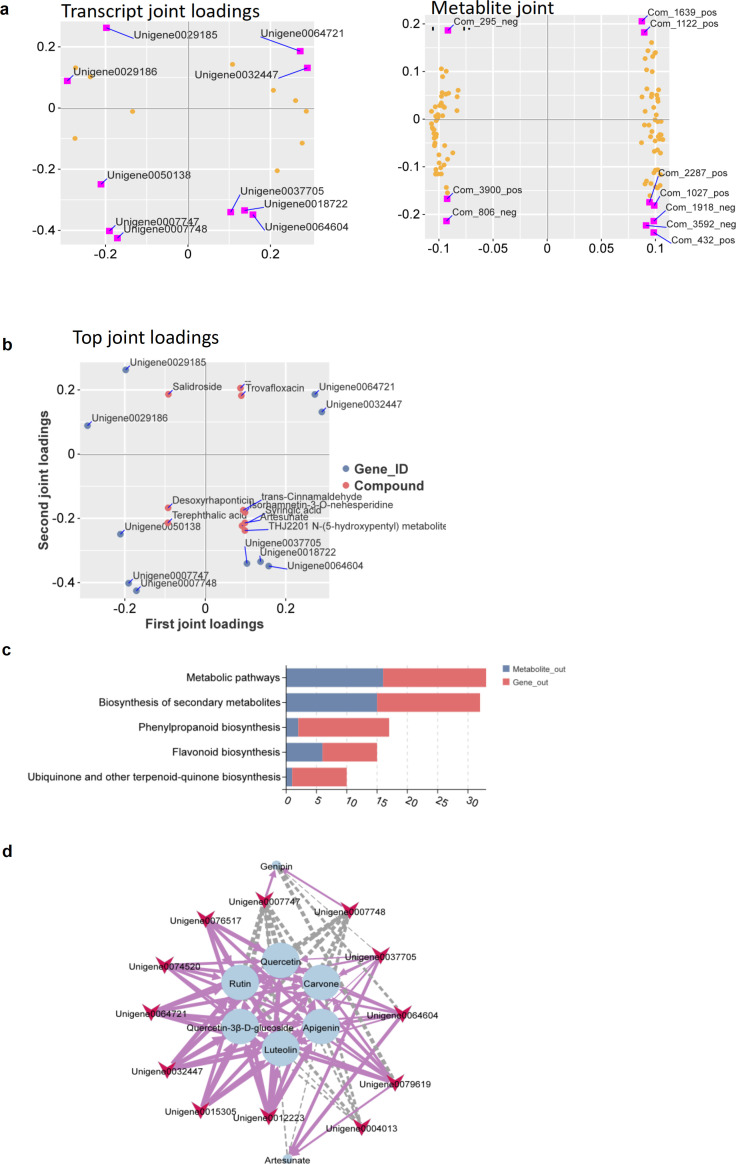
Integrated metabolome and transcriptome correlation analysis. **(a)** bidirectional orthogonal projections to latent structures (O2PLS) loadings plot showing the overall distribution and co-variation of the transcriptomic and metabolomic datasets. **(b)** Variable association loadings plots derived from the O2PLS model. The loading value reflects the contribution of each gene/metabolite to the intergroup differences between metabolite and gene. A positive or negative loading value indicates a positive or negative association with another omics component, respectively. **(c)** Integrated Kyoto Encyclopedia of Genes and Genomes (KEGG) pathway analysis illustrating the coordinated expression changes of functional genes and associated metabolites. **(d)** Gene–metabolite correlation network analysis based on Pearson’s correlation coefficients, constructed using the selected core differentially expressed genes (DEGs) and differentially accumulated metabolites (DAMs).

To elucidate the association between each metabolite and gene, loading plots were separately generated for different omics for certain associated variables (genes or metabolites) (**Figure 6a**). The loading value represents the contribution degrees of metabolites/genes in each component. Based on the results of the variation of DEGs and DAMs at 7 dpi, the top 10 genes and metabolites were selected to construct the loading map using the square of the loading values of the first two dimensions, which presented the most correlated genes and metabolites (**Figure 6b**). These metabolites participate in processes such as plant metabolic transformation, regulation of biological activity, and immune defense. DEGs and DAMs were mapped to the KEGG pathway database, including the biosynthesis of secondary metabolites, phenylpropanoid biosynthesis, and flavonoid biosynthesis (**Figure 6c**).

**Figure 7:**
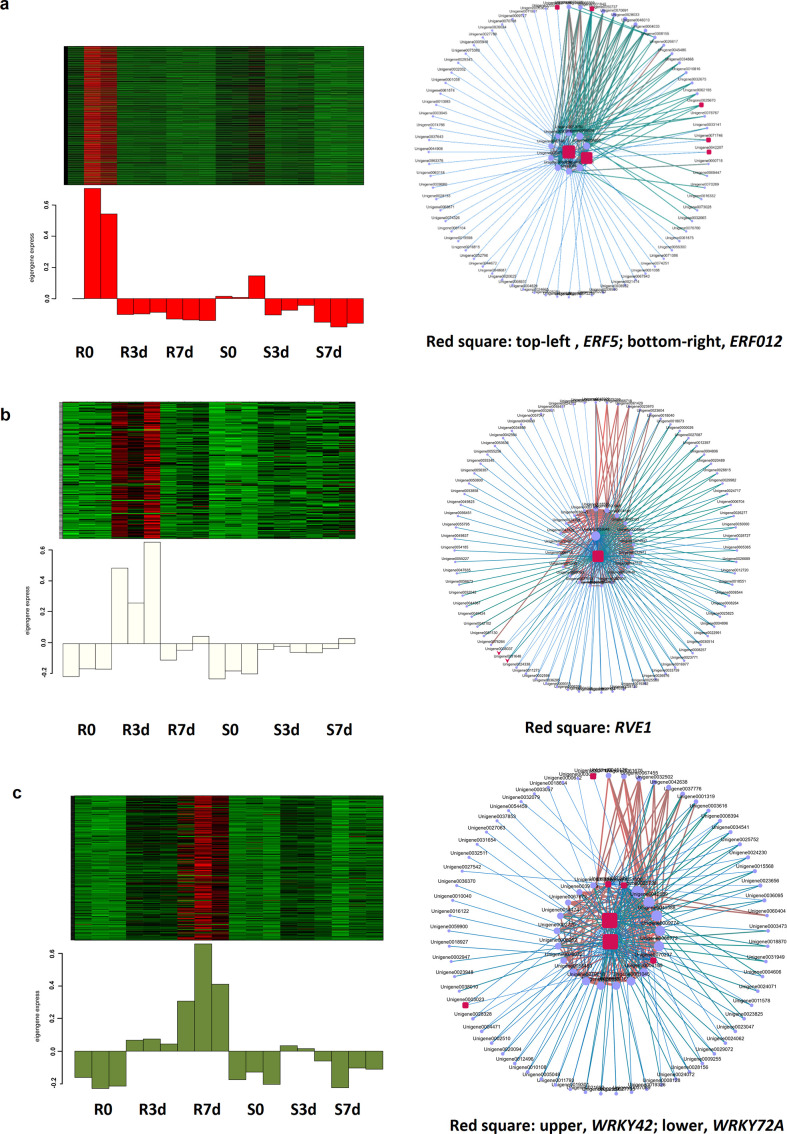
Weighted Gene Co-expression Network Analysis (WGCNA) of differentially expressed genes (DEGs) responsive to *Botryosphaeria dothidea* infection at 0, 3, and 7 dpi. **(a)** The red module comprising DEGs significantly associated with the early infection stage. **(b)** The floral white module comprising DEGs significantly associated with the middle infection stage. **(c)** The dark olive green module comprising DEGs significantly associated with the late infection stage. R represents the resistant *Osmanthus fragrans* accession No. 35, and S represents the susceptible accession No. 37. Infection stages are defined as early (0 dpi), middle (3 dpi), and late (7 dpi). For each module plot, the upper panel shows a heatmap of normalized expression levels of all genes in the module across all samples, with red indicating upregulated expression and green indicating downregulated expression; the lower panel displays a bar chart of the module eigengene (ME) values across different samples, reflecting the overall expression trend of the module. Hub genes for each module are marked with red squares on the right.

According to metabolomic results, a large number of flavonoids (e.g., apigenin and luteolin) were significantly enriched at R7d, and their biosynthesis-related genes, including *4CL* (4-coumarate-CoA ligase), a key enzyme in the phenylpropanoid pathway, and *F3H* (flavanone 3-hydroxylase), a key gene in the flavonoid synthesis pathway, were highly expressed in the transcriptome at this time point. To further dissect the regulatory relationships between these genes and flavonoid accumulation, we constructed a correlation network based on Pearson’s correlation coefficients (with the absolute Pearson’s correlation >0.5) for the 7-dpi samples (**Figure 7d**). This network revealed tight connections between flavonoid-related metabolites and their corresponding biosynthetic genes. The results expound that *4CL* (including 4CL-like genes) and *F3H* genes exhibited significantly positive correlations with flavonoid biosynthesis. For instance, the upregulated DEGs, *4CL3* and *F3H-2*, showed a strong positive correlation with the accumulation of luteolin (r = 0.91, p ≤ 0.01; r = 0.79, p ≤ 0.05, respectively) in R7d. *4CL-like9* showed a strong positive correlation with the accumulation of apigenin (r = 0.95, p ≤ 0.01). These results collectively indicate that the coordinated regulation of structural genes in flavonoid biosynthesis pathways forms the molecular basis for the metabolic reprogramming underlying resistance in *O. fragrans* against *B. dothidea* infection.

### Identification of gene co-expression modules during *B. dothidea* infection

To elucidate transcriptional networks in *O. fragrans* leaves during *B. dothidea* infection at 0, 3, and 7 dpi, we constructed co-expression networks associated with the defense response of *O. fragrans* to *B. dothidea* infection using RNA-seq data via Weighted Gene Co-expression Network Analysis (WGCNA). WGCNA identifies gene modules with similar expression patterns to investigate module–phenotype associations. Given the pivotal role of transcription factors (TFs) in regulating defense gene expression, DEGs were categorized into functional modules, and hub TFs underlying the transcriptional regulation of disease resistance were targetedly identified via co-expression networks.

In this study, 19, 874 genes were assigned to 17 co-expression modules. Time-resolved transcriptional profiling of *B. dothidea*-infected *O. fragrans* accessions revealed three modules exhibiting significant associations with distinct infection phases: early phase (0–1 h post-inoculation), asymptomatic, no visible lesions; middle phase (3 dpi), restricted lesions (diameter ≤ 2 mm) confined to inoculation sites; and late phase (7 dpi), progressive lesion expansion to leaf margins (lesions expanding to leaf margins in No. 37, >36% leaf area). Module–trait correlation analysis demonstrated strict temporal coordination between expression dynamics and symptom evolution.

WGCNA revealed that the red, flora-white, and dark-olive green modules were significantly correlated with defense responses between resistant (R) and susceptible (S) accessions at early (0 dpi), middle (3 dpi), and late (7 dpi) infection stages. Early stage (0 dpi): Genes in the red module were significantly upregulated in resistant materials, featuring TFs such as *ERF5* homolog (Unigene0075394) and *ERF012* homolog (Unigene0060514). These TFs correlated with BHLH41 (Unigene0001840) expression to regulate secondary metabolites, resistance genes, and hormone homeostasis (**Figure 7a**). Middle stage (3 dpi): The flora white module showed enhanced expression in the group R3 vs S3, *RVE1* homolog (Unigene0057706), a *MYB* factor family, identified as a key activator (**Figure 7**). Late stage (7 dpi): *WRKY42* (Unigene0041056) and *WRKY72A* (Unigene0039924) homologs enriched in the dark olive green module of R7 vs S7 (**Figure 7c**). Additionally, broad-spectrum resistance-associated TFs (e.g., *bZIP* and *GOLDEN2-LIKE/GLK*) enriched in this module, which were linked to secondary metabolism (flavonoids and terpenoids) and stress responses.

Collectively, *O. fragrans* establishes a layered immune defense network through stage-specific modules and TFs (*ERF*, *MYB*, *WRKY*, etc.), coordinating defense responses at each infection stage against *B. dothidea*.

## Discussion

*B. dothidea*, as a globally prevalent phytopathogen, poses a severe threat to economically valuable woody plants, including apples, pears, and kiwifruits. It induces leaf wilting and necrotic lesions and disrupts nutrient transport, ultimately leading to substantial post-harvest losses in agricultural production (Marsberg et al., 2017). Despite numerous studies on the resistance regulatory mechanisms against this pathogen in woody plants, including pear and Chinese hickory (Lv et al., 2025; Sun et al., 2024; Yang et al., 2024), the interplay between host phenotype and molecular responses during *B. dothidea* infection remains poorly characterized. This knowledge gap has hindered the genetic improvement of sweet osmanthus resistance, highlighting the urgent need for systematic investigations into its defense mechanisms.

To address this, we employed an integrated multi-omics approach combining transcriptome and metabolome analyses to dissect the early defense responses of sweet osmanthus leaves to *B. dothidea*. Resistant material No. 35 and susceptible material No. 37 were identified, and No. 35 exhibited restricted lesion expansion, while the necrotic area of No. 37 reached 42%.

Our findings reveal that the transcriptional regulatory networks underlying sweet osmanthus response to *B. dothidea* are inherently stage-specific, with distinct hub gene defense at different infection phases. During the early defense stage (0 dpi), the *AP2/ERF* family transcription factors *ERF5* and *ERF012* emerged as core regulators. *ERF* genes often function as substrates for MAPKs, forming synergistic or antergic disease resistance networks through phosphorylation modifications. Multiple studies have confirmed their conserved roles in modulating SA-mediated defense, PR gene expression, and pathogen resistance across plant species (Fischer and Dröge-Laser, 2004; Zheng et al., 2019; Wang et al., 2020). At the middle infection stage (3 dpi), MYB family member *RVE1* was the key hub gene. Previous studies have shown that *RVE1* participates in hormone signaling, secondary metabolism, and oxidative stress tolerance by regulating Reactive Oxygen Species (ROS) scavenging and cellular redox homeostasis (Lai et al., 2012; Nguyen and Lee, 2016; Abuelsoud et al., 2020). By the late infection stage (7 dpi), the phenotypic divergence between resistant and susceptible materials was more prominent, and *WRKY42* and *WRKY72A* act as hub genes. *WRKY*s are core regulators that induce callose deposition (Saberi Riseh et al., 2025; Pillai et al., 2018; Chowdhury et al., 2016) and are involved in SA/JA signal transduction during pathogen infection (Schuttenhofer et al., 2015; Wang et al., 2025; Zhang et al., 2023).

Beyond transcriptional regulation, sweet osmanthus resistance is closely linked to the stage-specific DAMs. At 3 and 7 dpi, significant differences in DAMs between resistant and susceptible materials formed a distinct response pattern: phenylpropanoid secondary metabolism initiated defense at 3 dpi, and flavonoid biosynthesis enhanced resistance at 7 dpi. This is consistent with well-established plant disease resistance mechanisms, where phenylpropanoids and flavonoids serve as core defensive metabolites with antimicrobial activity and ROS-scavenging properties (Li et al., 2025; Kumar et al., 2020a, Kumar Patel et al., 2020b; Hu et al., 2024). Notably, in susceptible material No. 37, the double transcriptional dysregulation of SA biosynthesis and inactivation pathways resulted in insufficient active SA in the susceptible *O. fragrans* accession at 7 dpi. This supports the critical role of SA in plant defense against biotrophic and hemibiotrophic pathogens, where its adequate accumulation is essential for activating downstream resistance responses.

This study supposed flavonoid biosynthesis as the core disease resistance mechanism in sweet osmanthus, as key flavonoid biosynthesis genes *4CL* and *F3H* were significantly upregulated in the resistant material No. 35 at 7 dpi, accompanied by high enrichment of flavonoids, including luteolin and apigenin. Flavonoids have been widely confirmed to exert direct antimicrobial activity, thus showing great potential as eco-friendly biological control alternatives to chemical pesticides. Unlike SA biosynthesis-dependent resistance in apple and lignin biosynthesis-dependent resistance in pear, we hypothesize that *WRKY42* and *WRKY72A* enhance sweet osmanthus resistance to *B. dothidea* by regulating flavonoid biosynthesis, which provides theoretical support for our hypothesis by previous reports that WRKY transcription factors modulate flavonoid metabolism (Hu et al., 2020; Zhang et al., 2023).

In conclusion, this study systematically clarifies the molecular and metabolic mechanisms underlying *O. fragrans* resistance to *B. dothidea* via integrated multi-omics analysis. *O. fragrans*, a woody aromatic plant, harbors abundant flavonoid precursors in leaves and adopts a unique metabolic defense strategy. It preferentially establishes a chemical defensive barrier through rapid accumulation of defensive flavonoids by upregulating key biosynthetic genes, rather than relying on hormone signal amplification to initiate defense responses.

To our knowledge, this is the first systematic multi-omics study on the *O. fragrans*–*B. dothidea* interaction mechanism. We further uncover a sequential defense regulatory network mediated by stage-specific ERF, RVE, and WRKY transcription factors, coordinated with defensive metabolite accumulation. These findings not only deepen our understanding of woody plant–pathogen interactions but also offer a valuable reference for defense mechanism studies in other woody plant–pathogen systems.

Several key research directions remain to be explored in future work: 1) functional validation of the identified hub transcription factors (*ERF5*, *ERF012*, *RVE1*, *WRKY42*, and *WRKY72A*) and core flavonoid biosynthetic genes (*4CL* and *F3H*) via genetic transformation, 2) field efficacy trials of flavonoid-based resistance inducers to evaluate their practical application potential, and 3) exploration of the crosstalk between transcriptional regulators and defensive metabolite biosynthesis to fully delineate the sweet osmanthus defense network.

## Materials and methods

### Plant, isolate materials, and inoculation

*O. fragrans* germplasms were provided by the Institute of Garden Plants and Flowers (Zhejiang Academy of Agricultural Sciences, Zhejiang, China). The plant materials were cultivated in a natural environment and sequentially numbered. Branches with leaves were cut off, transferred to the laboratory, surface-disinfected with 75% ethyl alcohol, and rinsed twice with sterile distilled water. The prepared leaf-bearing branches were wrapped with wet absorbent cotton to maintain humidity.

Pathogenic fungi were isolated from *O. fragrans* leaves with typical leaf spot symptoms and identified as *B. dothidea*. Purified isolates were inoculated onto Potato Dextrose Agar (PDA) medium and incubated at 28 °C for 7 days. Mycelial plugs (0.5-cm diameter) were excised from 7-day-old PDA cultures and detached *O. fragrans* leaves (pre-wounded site). Inoculated leaves were incubated at 28 °C for 7 days, with infection sites sampled at 0, 3, and 7 dpi for RNA sequencing. The 0-dpi control samples were collected 30 minutes after inoculation. Three biological replicates were performed for each treatment.

### Plant sensitivity analysis

Fifty *O. fragrans* germplasms preserved at the Institute of Garden Plants and Flowers were employed for an initial screening of pathogenic sensitivity via inoculation. Inoculated leaves were incubated at 28 °C under humid conditions for 7 days, with daily monitoring of symptom appearance and development. Symptoms on branches were evaluated based on the size and quantity of lesions across all infected sites. Infection type was rated on a progressive scale (0–3): 0 = no visible infection; 1 = small lesions, 0.5 cm, equivalent to the size of mycelial plugs; 2 = medium lesions (2–3 cm); and 3 = large lesions, more than 3 cm (>3 cm, extending to leaf margins). Additionally, the frequency of each infection type per accession was quantified.

### Transcriptome analysis

#### RNA isolation and library preparation

Total RNA from *O. fragrans* leaves was extracted using the TRIzol reagent kit (Invitrogen, Carlsbad, CA, USA) according to protocol, the RNA quality was evaluated using the Agilent 2100 Bioanalyzer (Agilent Technologies, Palo Alto, CA, USA), and a cDNA library was constructed with random primers, purified using the QiaQuick PCR extraction kit (Qiagen, Venlo, The Netherlands), and sequenced on an Illumina platform (Illumina NovaSeq 6000) by Gene Denovo Biotechnology Co. (Guangzhou, China). In order to obtain high-quality clean reads, raw reads were filtered using fastp (version 0.18.0; Chen et al., 2018). *De novo* transcriptome assembly was performed using the short-read assembly program Trinity (Grabherr et al., 2011). Unigene expression levels were calculated and normalized to FPKM (Mortazavi et al., 2008). The cDNA and small RNA libraries were sequenced using Illumina NovaSeq 6000 by Gene Denovo Biotechnology Co. (Guangzhou, China).

### Differentially expressed gene analysis

PCA and DEG analysis were performed using the R package stats (http://www.r-project.org/) and DESeq2 software (Love et al., 2014) for intergroup comparisons and edgeR (Robinson et al., 2010) for pairwise sample comparisons. Genes with a false discovery rate (FDR) <0.05 and |log_2_(fold change, FC)| ≥ 2 were defined as DEGs. All DEGs were annotated to GO terms (http://www.geneontology.org/) and mapped to KEGG pathways (Kanehisa and Goto, 2000), with the number of genes per term/pathway calculated. p-Values were subjected to FDR correction, and GO terms/pathways with an FDR ≤ 0.05 were considered significantly enriched in DEGs.

### *O. fragrans* leaf metabolomics analysis

Leaf tissues (100 mg) of *O. fragrans* were rapidly frozen in liquid nitrogen, and the homogenate was resuspended in 500 μL of prechilled 80% methanol and vortexed. Part of the supernatant was diluted with Liquid Chromatography-Mass Spectrometry (LC-MS)-grade water to a final methanol concentration of 53%. The supernatant was added to an Liquid Chromatography-Tandem Mass Spectrometry (LC-MS/MS) system (Want et al., 2012). Raw UHPLC–MS/MS data files were subjected to processing in Compound Discoverer 3.1 (CD3.1, Thermo Fisher, Hesse, Germany) for the purposes of peak alignment, peak picking, and metabolite quantitation. Ion peaks were predicted using normalized data, and these predicted peaks were then matched against the mzCloud (https://www.mzcloud.org/), mz Vault, and Mass List databases to secure accurate qualitative and relative quantitative outcomes. These reliable data were subjected to multivariate statistical analysis, differential metabolite analysis, pathway analysis, and trend analysis. DAMs were filtered based on the VIP score, which is used to represent the importance of variables and their contributions in sample discrimination from the orthogonal partial least squares discriminant analysis (OPLS-DA) model for group discrimination (p-value of *t*-test <0.05 and VIP ≥ 1).

### Correlation analysis

Based on gene expression level and metabolite accumulation, a correlation analysis between DEGs and DAMs was performed and analyzed using three models, such as the pathway model, the O2PLS model, and Pearson’s model, to decipher the association of transcript and metabolite data. All differentially expressed genes and metabolites at 7 dpi were mapped to the KEGG pathway database to obtain their links in metabolic pathways. O2PLS models were calculated using the OmicsPLS package of R (El Bouhaddani et al., 2016). The selected genes and metabolites were used for heatmap analysis using the pheatmap package in the R project (Kolde, 2015). Additionally, these genes and metabolites (with the absolute Pearson’s correlation >0.5) were applied for metabolite transcript network analysis using the igraph package in the R project (Csardi, 2006).

### Co-expression network analysis for the construction of modules

The WGCNA (v1.47) R package (Langfelder and Horvath, 2008) was employed to construct co-expression networks, with the aim of identifying highly co-expressed gene clusters (modules) and relating these modules to phenotypic traits of *O. fragrans* leaves. Correlation analysis was performed by evaluating the association between module eigengenes and specific trait/phenotype datasets. Pearson’s correlation coefficients between individual genes and data within the module were calculated, as well as the relevant modules (defined by positive or negative relevance) corresponding to each phenotype. For genes within each module, GO term and KEGG pathway enrichment analyses were carried out to characterize their biological functions.

### RT-PCR validation

Total RNA from *O. fragrans* leaves was extracted using the TRIzol reagent kit (Invitrogen, Carlsbad, CA, USA) according to protocol. The yield of RNA was determined using a NanoDrop 2000 spectrophotometer (Thermo Scientific, USA). Real-time PCR was performed using the LightCycler^®^ 480 II Real-Time PCR System (Roche, Switzerland). Primer sequences are detailed in **Supplementary Table 8**. The relative expression levels of *CIPK7*, *CPK1*, *WRKY46*, *XA21*, *Fls2*, and *LEA46* were normalized to the reference gene *ACT* and were quantified using the 2^−ΔΔCt^ method (Livak and Schmittgen, 2001). All reactions were performed with three technical replicates per sample, and three independent biological replicates were included for each treatment.

## Data Availability

The raw data have been deposited in the NCBI Sequence Read Archive (SRA) database under the BioProject accession number PRJNA1312151.

## Supplementary material

Supplementary Figure 1 Transcriptional dynamics of DEGs in two *O. fragrans* accessions in response to *B. dothidea* infection.

Supplementary Figure 2 The DAMs variation in two *O. fragrans* leaves response to *B. dothidea* infection in R0 VS S0.
