# Supplementary material for: Integrated multi-omics analysis revealed the response mechanism of Osmanthus fragrans leaves against the infection by Botryosphaeria dothidea
Source: Front Plant Sci. 2026 May 15;17:1830904. doi: 10.3389/fpls.2026.1830904 (PMC13219255; doi:10.3389/fpls.2026.1830904)
Supplement: Supplementary Figure 1 — Transcriptional dynamics of DEGs in two O. fragrans accessions in response to B. dothidea infection. [file Image1.pdf]

a R0d vs S0d

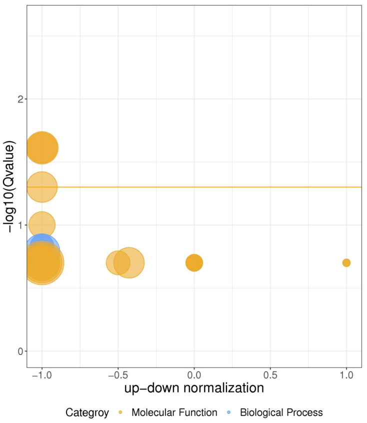

ID	Descrption
GO:0016705	oxidoreductase activity, acting on paired donors, with incorporation or reduction of molecular oxygen
GO:0005506	iron ion binding
GO:0020037	heme binding
GO:0046906	tetrapyrrole binding
GO:0004497	monooxygenase activity
GO:0048544	recognition of pollen
GO:0009875	pollen−pistil interaction
GO:0008037	cell recognition
GO:0006952	defense response
GO:0008447	L−ascorbate oxidase activity
GO:0003854	3−beta−hydroxy−delta5−steroid dehydrogenase activity
GO:0033764	steroid dehydrogenase activity, acting on the CH−OH group of donors, NAD or NADP as acceptor
GO:0016229	steroid dehydrogenase activity
GO:0047622	adenosine nucleosidase activity
GO:0072585	xanthosine nucleotidase activity
GO:0016491	oxidoreductase activity
GO:0003700	DNA−binding transcription factor activity
GO:0016630	protochlorophyllide reductase activity
GO:0042409	caffeoyl−CoA O−methyltransferase activity
GO:0016709	oxidoreductase activity, acting on paired donors, with incorporation or reduction of molecular oxygen, NAD(P)H as one do ...

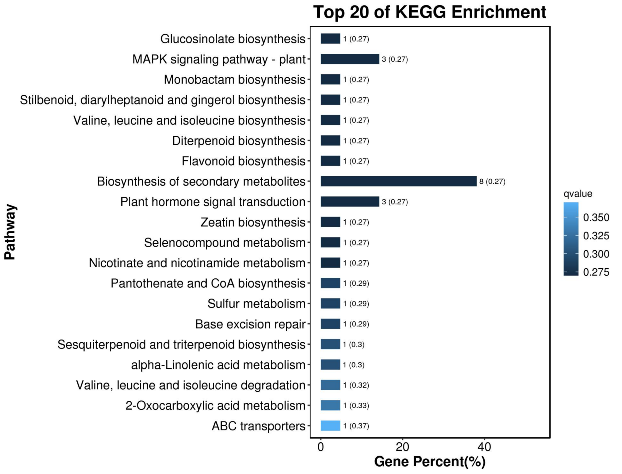

b R3d vs S3d

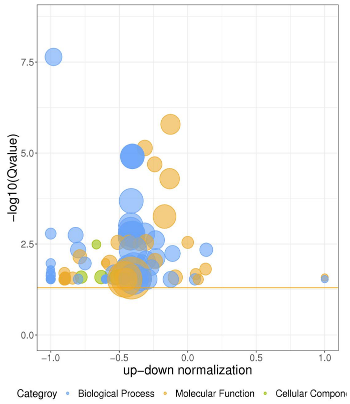

ID	Descrption
GO:0015074	DNA integration
GO:0003700	DNA−binding transcription factor activity
GO:0046906	tetrapyrrole binding
GO:0006355	regulation of transcription, DNA−templated
GO:1903506	regulation of nucleic acid−templated transcription
GO:2001141	regulation of RNA biosynthetic process
GO:0020037	heme binding
GO:0140110	transcription regulator activity
GO:0051252	regulation of RNA metabolic process
GO:0016491	oxidoreductase activity
GO:2000112	regulation of cellular macromolecule biosynthetic process
GO:0010556	regulation of macromolecule biosynthetic process
GO:0005975	carbohydrate metabolic process
GO:0009637	response to blue light
GO:0006351	transcription, DNA−templated
GO:0097659	nucleic acid−templated transcription
GO:0031326	regulation of cellular biosynthetic process
GO:0019219	regulation of nucleobase−containing compound metabolic process
GO:0009416	response to light stimulus
GO:0032774	RNA biosynthetic process

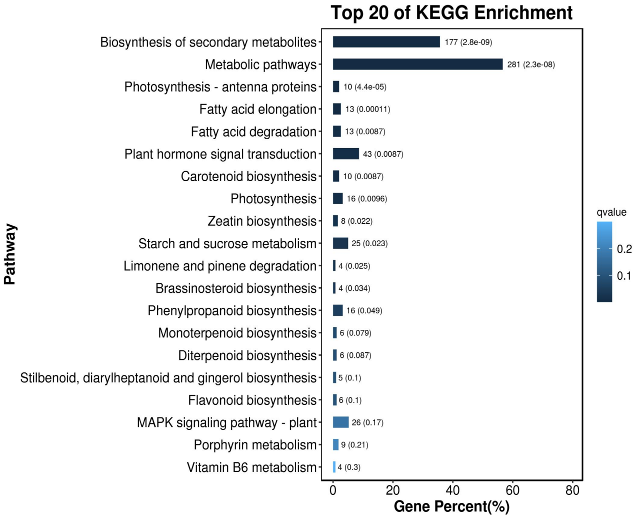

c S0 vs S7d

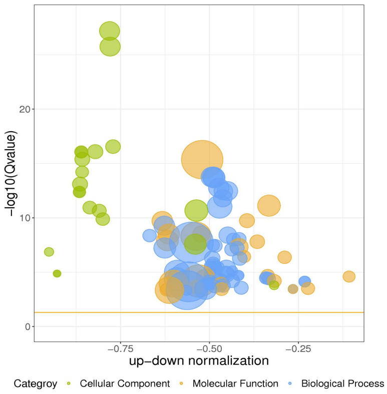

ID	Descrption
GO:0009507	chloroplast
GO:0009536	plastid
GO:0009579	thylakoid
GO:0009526	plastid envelope
GO:0009534	chloroplast thylakoid
GO:0031976	plastid thylakoid
GO:0009570	chloroplast stroma
GO:0003824	catalytic activity
GO:0042651	thylakoid membrane
GO:0019752	carboxylic acid metabolic process
GO:0043436	oxoacid metabolic process
GO:0006082	organic acid metabolic process
GO:0009532	plastid stroma
GO:0005975	carbohydrate metabolic process
GO:0044283	small molecule biosynthetic process
GO:0016053	organic acid biosynthetic process
GO:0009535	chloroplast thylakoid membrane
GO:0055035	plastid thylakoid membrane
GO:0046394	carboxylic acid biosynthetic process
GO:0016491	oxidoreductase activity

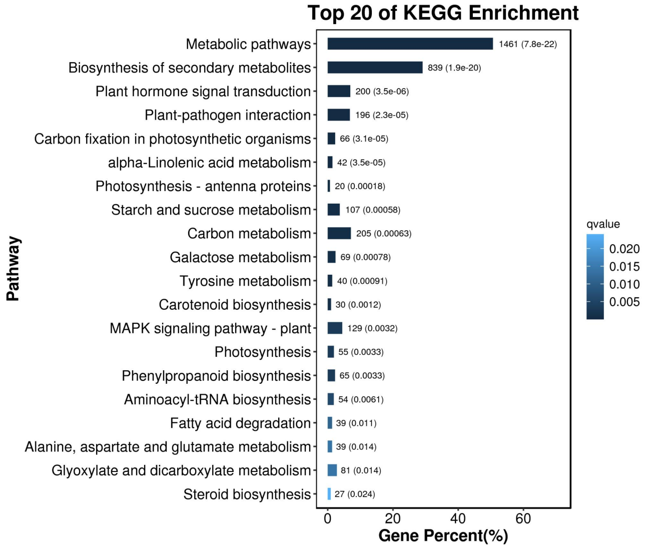

Figure S1. Transcriptional dynamics of DEGs in two *O. fragrans* accessions in response to *B. dothidea* infection.

a. Bubble plot of the top 20 significantly enriched GO terms (left) and bar plots of the top 20 significantly enriched KEGG pathway(right) for the R0d vs S0d comparison. b. Bubble plot of the top 20 significantly enriched GO terms(left) and bar plots of the top 20 significantly enriched KEGG pathway(right) for the R3d vs S3d comparison. c. Bubble plot of the top 20 significantly enriched GO terms(left) and bar plots of the top 20 significantly enriched KEGG pathway(right) for the S0d vs S7d comparison.

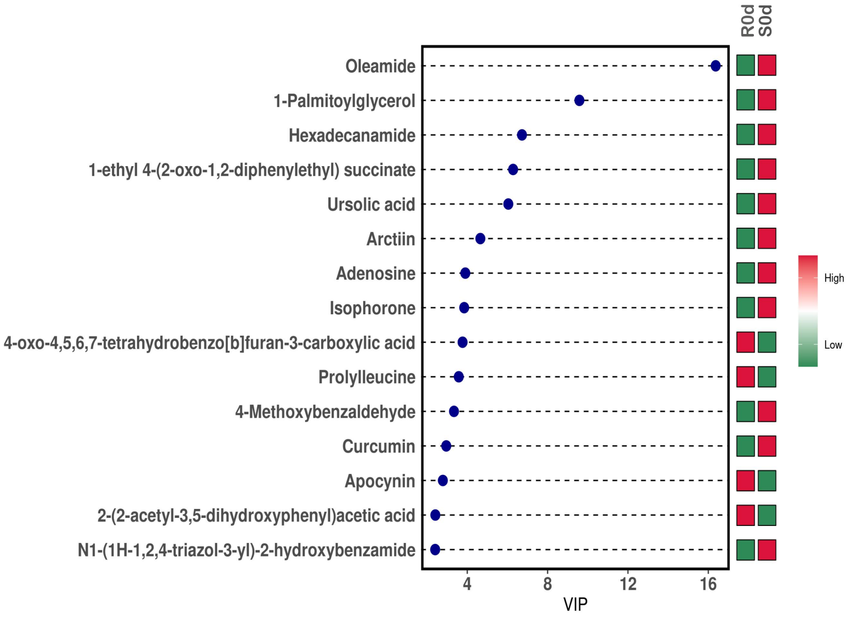

Figure S2. The DAMs variation in two *O. fragrans* leaves responce to *B. dothidea* infection in R0 VS S0.

R represents the resistant *O. fragrans* accession No. 35, and S represents the susceptible *O. fragrans* accession No. 37. The number following R/S indicates the sampling time point (0 d, 3 d, 7 d) days post inoculation (dpi). Orthogonal Partial Least Squares Discriminant Analysis (OPLS-DA) was performed to identify metabolites contributing significantly to intergroup differences. Red indicates abundance accumulation,and green indicates Less accumulation.
